# In Search of Safe Havens: Identifying priority conservation areas for a critically endangered bird, the Great Indian Bustard, in the Deccan Landscape of India

**DOI:** 10.1371/journal.pone.0355780

**Published:** 2026-08-14

**Authors:** Shaheer Khan, Gautam Talukdar, Bilal Habib

**Affiliations:** Wildlife Institute of India, Dehradun, Uttarakhand, India; Zoological Survey of India, INDIA

## Abstract

The prevention of local extirpation and restoration of populations across their ranges are always considered central conservation goals, especially for species that are tagged as endangered. Many species of different taxa face the risk of extinction due to various threats and require urgent management interventions. Protecting and conserving such species in their native area requires multiple methods and management strategies, such as identifying priority conservation areas. The Great Indian Bustard *Ardeotis nigriceps*, a critically endangered species per the IUCN Redlist, was once found throughout India and southern Pakistan. It is now confined to very few patches, with 100–150 individuals left in the wild. Though they are critically endangered, their habitat requirements are poorly understood, hindering their conservation. This paper modelled a probability distribution map and identified priority conservation areas for the GIB in the Deccan landscape. The study found that out of the 7,20,000 km^2^ Area of the Deccan Landscape, only 48,000 km^2^ (6.6%) of the area was found suitable for the species using various environmental and climatic variables. We identified 16 critical sites that totalled 18,000 km^2^, ranging from 144 to 8496 km^2^, as priority conservation areas by conducting a landscape-level survey and species distribution modelling. The potential conservation areas are a mosaic of grassland and agricultural fields. The future of such areas will depend on the revival of traditional farming systems and on maintaining the ecological integrity of remnant grassland patches. Restoring habitat and traditional farming patterns in these areas is recommended to protect the species from extinction. This study aims to contribute to the management of GIB by identifying the extent of its potential habitat and high-priority conservation areas to ensure the species’ survival in the southernmost part of its distribution.

## Introduction

India is a globally important biodiversity region that contributes 2.4% of the total world geographic area, and still, its contribution to the overall world global diversity is around 8% of species [1,2]. Moreover, it harbours nearly 1.46 billion people (worldometer.info) and is experiencing rapid infrastructure development and other anthropogenic pressures that directly or indirectly affect species. This anthropogenic expansion leads to the extirpation of the species from their native ranges, and the effect is more pronounced for the species that share their habitat with humans [3,4]. Many species of different taxa face the risk of extinction due to various threats and require urgent management interventions to restore the thriving population across their historical ranges [5–7]. The prevention of local extirpation and restoration of populations across their ranges are central conservation goals, especially for the species that are tagged as endangered [8].

Protecting and conserving species in their native area requires various methods and management strategies, such as identifying priority conservation areas and creating protected areas (PAs). The PAs are essential for biodiversity conservation, providing habitat and protection for the concerned species by prohibiting anthropogenic activities from maintaining ecological processes [9,10]. However, land sharing between local communities and wildlife is commonly supported in areas where the PA network is insufficient to meet conservation targets [11]. Moreover, forming PAs to conserve species that utilise large areas and share the landscape with humans is not feasible. Identifying priority conservation areas is more reasonable in the context of species thriving in human-dominated landscapes and is crucial in landscape-level conservation management to enhance habitat connectivity and increase gene flow [12]. However, a thorough study is required to identify these areas to understand the species’ ecology and habitat requirements, which implies management interventions to maximise survival by conserving potential habitat patches [13].

In India, many species face the risk of extinction due to various threats and require urgent management interventions [5,14]. One such species at a high risk of extinction and needing immediate conservation management is critically endangered, the Great Indian Bustard *Ardeotis nigriceps* (hereafter GIB). The GIB is a large, ground-dwelling bird primarily found in the arid and semi-arid grasslands of India. It is an omnivore, feeding on seeds, insects, small reptiles, and rodents. While often observed solitarily or in small groups, they may form larger flocks seasonally. The species follows a polygynous breeding system, where males perform elaborate courtship displays to attract multiple females. Nesting occurs on the ground, with females solely responsible for incubation and chick-rearing. The birds were once found in 11 states in India and parts of Pakistan in large flocks across the grasslands, but the population went down rapidly at the end of the 19th century [15,16]. The most current estimate places the population in a range between 100 and 150 individuals [17]. However, this population range is wide for critically endangered species, which impedes the implementation of any conservation measures [18]. The largest population of GIB is found in Rajasthan [19], and the remaining states, Gujarat, Madhya Pradesh, Maharashtra, Karnataka, and Andhra Pradesh, have 10–15 individuals [20]. The bird has completely disappeared from the northern parts of India, and the current populations of Maharashtra, Karnataka, Telangana, and Andhra Pradesh (Deccan landscape) are at a high risk of local extinction. Studies have documented a sharp population decline, with the Deccan landscape identified as one of the most severely affected regions [18]. The previous GIB estimates in the landscape were 200–390 individuals in 1989 [16], which decreased to 66–75 individuals in 2008 [21,22].

Furthermore, a systematic landscape-level survey in Maharashtra estimated fewer than eight individuals in the state [23]. The decrease in their number is mainly due to habitat depletion, rapidly growing irrigation networks, changes in traditional agricultural practices, and an increase in human activities in GIB-bearing areas [24–26]. The landscape is experiencing rapid conversion of agricultural lands and a significant loss of grassland habitats has been observed due to various anthropogenic factors [27]. In addition, farmers have been shifting to cash crop production from the traditional farming of pulses and oilseeds [28,29]. The conversion of grasslands and shrublands into agricultural land, along with their use for plantation drives, has made these ecosystems prime targets for development projects [30]. Excessive use of insecticides and pesticides, overgrazing, urbanisation, feral dog population, and powerline expansion are also contributing to the decline of GIB populations. The genetic study on GIB also suggested extremely low genetic variability and a strong population bottleneck [31]. The present population is small and in isolated pockets. This isolation will lead to inbreeding depression and could result in local extinction. Larger and more connected populations are needed to safeguard GIB in the future. For the conservation of the species, identifying areas where intensive conservation measures should be implemented is essential to conserving the species and habitat to ensure GIB survival.

Species distribution modelling (SDM) is a powerful tool used to predict and understand the geographic distribution of species based on environmental conditions and species occurrence data [32,33]. When applied to critically endangered species, SDM offers several strengths and weaknesses, which are crucial to consider for effective conservation planning and management. SDMs can identify potential habitats for critically endangered species, even in areas where they have not been previously recorded [34]. This is essential for discovering unknown populations and prioritizing areas for conservation. SDMs integrate various types of data, such as climatic, topographic, and land-use data, providing a holistic view of the factors influencing species distributions. They allow analysis at different spatial and temporal scales, accommodating the varying ecological needs of critically endangered species. SDMs help in identifying critical habitats that need protection and guiding the establishment of site-specific conservation measures [35–38]. By highlighting key habitats, SDMs assist in the efficient allocation of limited conservation resources. The models can assess the impact of potential threats such as habitat fragmentation, climate change, and human activities, enabling proactive management strategies.

However, critically endangered species often have limited occurrence records, leading to challenges in building accurate models. This scarcity of data can result in unreliable predictions. Validating SDMs for critically endangered species is difficult due to the limited availability of independent data for comparison and the ethical concerns of further stressing small populations for validation purposes. Implementing conservation strategies based on SDMs can be hindered by socioeconomic factors, such as land ownership, local community needs, and political considerations.

The MaxEnt models have become a popular SDM tool to model the potential distribution of rare or threatened species of conservation concern [39,40] and are also used to improve the understanding of ecological factors for conservation planning [32,41]. This study used the MaxEnt modelling for GIB in the Deccan landscape to address multiple conservation goals, explicitly identifying potential distribution and priority conservation areas. We predicted the potential distribution map using GPS-tagged individual data from 2013 to 2015. Although the dataset dates back to 2013–2015, it remains important because the GIB’s very low numbers make such movement information rare and invaluable for predicting its potential distribution. GPS data provide precise and accurate locations, which are crucial for modelling the distribution of critically endangered species [42]. This precision helps in identifying key habitats and fine-scale habitat use [43]. Moreover, it reduces human error and observer bias, providing more reliable data.

Our result provides an overview of potential habitats available for GIB in the Deccan landscape. We further verified the potential area by conducting a landscape-level survey in Maharashtra for GIB presence using distance sampling and questionnaire surveys. Based on the survey and distribution modelling, we identified areas where the species’ presence is confirmed and should be considered for protection to ensure GIB survival and future conservation. Our overarching aim was to identify areas that provide suitable habitat for GIB and thus help restore the species across its distribution range. Specifically, our objectives were to (1) map suitable habitats in the Deccan landscape based on the GPS location of tagged individuals (2) identify priority conservation areas, and validate the potential distribution map through a landscape-level survey. The final purpose of this study is to contribute to the conservation management of GIB by identifying the extent of its potential habitat and high-priority areas to ensure the species’ survival in the southernmost area of its distribution. Replicating such studies for species facing similar threats and dependent on ex-situ conservation involves adapting methodologies to diverse ecosystems, understanding the use of key variables for better prediction, and fostering collaborative efforts for knowledge sharing. Understanding local ecological factors and the interconnections between species is crucial.

## Materials and methods

### Study area

The Deccan landscape is a large plateau in western and southern India. It rises to 100 meters in the north and more than 1,000 meters in the south, forming a raised triangle within the south-pointing triangle of the Indian subcontinent’s coastline. It extends over eight Indian states and encompasses a wide variety of habitats, and the majority of the landscape is covered by Telangana, Maharashtra, Karnataka, and Andhra Pradesh states. The region’s climate varies from semi-arid in the north to tropical in most areas, with distinct wet and dry seasons. Rainfall generally occurs from June to October. The landscape is a semi-arid region of India that receives significantly less rain than other parts of the country, making it suitable for GIB. The summer season is dry and extremely hot, regularly exceeding 40 °C. The terrain gently undulates with mild slopes and flat-topped hillocks with intermittent shallow valleys forming the major drainage channels. Grassland areas are distributed in disjunct, fragmented patches, creating a mosaic of grazing land, agricultural land, and human settlements.

### Tagging and collection of occurrence data for GIB

Bird capture and tagging were conducted following approved protocols under permits issued by the Ministry of Environment, Forest and Climate Change (MoEFCC), Government of India, and the Maharashtra Forest Department (Permit No. SPP-01(2015), dated 08 April 2015).

**Fig 1:**
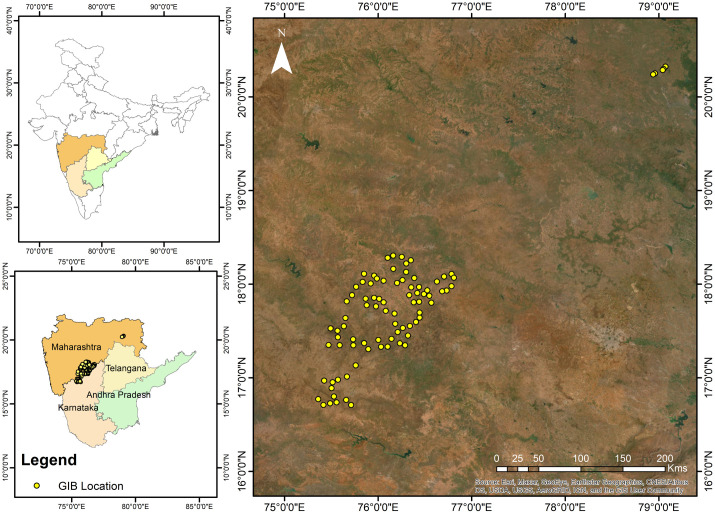
The presence location of GIB obtained and used in developing a potential distribution map from the three tagged individuals of GIB during 2013-2016. Basemap source: Esri, Maxar, GeoEye, Earthstar Geographics, CNES/Airbus DS, USDA, USGS, AeroGRID, IGN, and the GIS User Community.

We captured three individuals using noose traps and tagged them with Solar Argos/GPS PTT of 70 grams of weight (Microwave Telemetry Inc.) to identify the potential distribution and priority conservation areas for GIB in the landscape. The instrument recorded GPS locations daily at 00:00, 02:00, 04:00, 06:00, 12:00, 14:00, and 16:00 hrs. A comprehensive dataset of GIB occurrence points (2975 GPS locations) from three tagged individuals was collected from Oct 2013 to June 2016. The GPS points were in clusters as the GIBs are large birds and generally forage in small patches throughout the day [44]. To remove the spatial autocorrelation from the locations, we selected one GPS location from a 12*12 km grid and filtered 100 locations for distribution modelling (Fig 1). The selected grid size is biologically informed and also used in previous landscape-level GIB surveys in India [23,45]. So, to make it consistent, we used this grid size to allow consistency.

### Mapping of potential distribution area using a species distribution model

For mapping and identifying suitable GIB habitats throughout the Deccan landscape, we used maximum entropy modelling (MaxEnt) [46]. MaxEnt is based on a machine learning response intended to predict suitable habitats using presence-only data. This method estimates the most uniform distribution (maximum entropy) of sampling points compared to background locations, given the limitations derived from the data [47].

a) **Environmental data used in the analysis**

The bioclimatic variables were acquired from the Worldclim dataset version 2.0 [48]. MODIS NDVI 16-day composite grid data (MOD13Q1) were obtained for 2013–14 from the NASA Earth Observing System (EOS) data gateway. In addition, the global human population data for 2015 were acquired from Global Mosaics 2000–2020 data [49], distance from the powerline, global human footprint data, and land use land cover (LULC) data (S1 Table in S1 File). We did a Pearson’s correlation test between the 46 variables (climatic, environmental, and anthropogenic layers) before species distribution modelling and eliminated highly correlated variables (>0.6). Thus, we finalised 18 variables for final analyses (S1 Table in S1 File).

We resampled all the variables with a resolution of 30 arc seconds (~1 km) and subset them using the state boundary shapefile of Maharashtra, Karnataka, Andhra Pradesh, and Telangana. The final layers were converted into ASCII grid file format. All the analyses were done in ArcGIS 10.8.1 and MaxEnt software, version 3.3.3k (https://www.cs.princeton.edu/schapire/maxent) [46].

b) **Simulation procedure**

GIB presence data and selected variables were adapted to the format required by MaxEnt software [46]. Ten individual MaxEnt models were run in batch mode with the following settings: Auto features (feature types are automatically selected depending on the training sample size), logistic output format, replicates = 10, regularisation multiplier = 1, maximum iterations = 500, convergence threshold = 0.00001. We used a maximum of 1000 background points, generated using the Local Convex Hull (LoCoH) method in MaxEnt. The LoCoH algorithm constructs a spatially explicit boundary around the observed occurrence locations and restricts background sampling to this biologically relevant area. This approach reduces extrapolation into unsuitable or unsampled regions, thereby enhancing the ecological realism and robustness of the species distribution model [47]. We used the mean probabilities of the ten independent models predicted as estimates for subsequent analyses. We applied the bias correction method to account for sampling bias by creating bias files using the SDM tool in ArcGIS 10.8.1 [50,51]. In a biased file, the cell values reflect the sampling effort and give weight to random background data used for modelling [52]. Previous studies have shown that correcting sampling bias has yielded improved model fitting, especially with smaller sample sizes [52]. The spatial distance used to quantify the region of spatial bias was kept at 50 km. Model performance was measured by the area under the receiver operating characteristic curve (AUC) and True Skill Statistics (TSS) [46,53]. An AUC value of about 0.5 suggested that the distribution model is no better than random in predicting effects. The distribution model with a value above 0.7 is good and highly accepted. The TSS (sensitivity + specificity – 1) statistically depends on a threshold within a range of −1–1 [54]. A negative or close to 0 TSS value meant that the distribution model was random, whereas the TSS value of +1 suggested good performance of the distribution model. The results with a 100 km radius bias file were eliminated based on the poor AUC values and TSS score.

### Identification of priority conservation areas

A landscape-level survey was conducted in Maharashtra in 2017 to validate the potential distribution map and identify priority conservation areas for GIB. 372 grids of 12 x 12 km were laid in Maharashtra to identify potential areas. A total of 20 km of vehicle-based transects were sampled in each grid by 31 teams. Each team covered 12 grids on six consecutive days, one grid in the morning (0600-1000h) and one in the evening (1600-1900h), to collect the data when bird activity is expected to be highest. The teams surveyed their respective grids simultaneously to avoid double-counting a single individual. The information on the recent sighting of the bird was also collected through a semi-structured questionnaire survey of the local people from different villages within each grid (S2 Table in S1 File). The sighting of the GIB was confirmed by presenting each respondent with images of similar species in the landscape, specifically the woolly-necked stork (*Ciconia episcopus*), for comparison. Limited ecological knowledge of a species within a landscape remains a major constraint for the conservation of endangered species [55]. In such contexts, secondary sources of information, particularly local ecological knowledge, serve as valuable and often indispensable data inputs [56]. By combining the information about the bird through a questionnaire survey and species distribution modelling output. We identified priority conservation areas for GIB in the landscape.

### Comparison between priority conservation areas and random sites

To assess differences in land use and land cover (LULC) characteristics between priority conservation areas and randomly selected sites, we conducted a comparative analysis using LULC data. We selected 125 random sites across the study region to serve as a reference for comparison against the identified priority conservation areas. For each site, we extracted the proportion of different LULC categories.

To statistically evaluate the differences in LULC composition between the priority conservation areas and random sites, we performed an independent t-test. This test allowed us to determine whether the mean proportions of specific land cover types (e.g., fallow lands, crop types, grasslands, and water bodies) significantly differed between the two groups. The results provide insights into the distinct landscape characteristics of priority conservation areas and their potential ecological significance compared to the surrounding landscape.

## Results

### Potential habitat for GIB using species distribution modelling in the landscape

**Fig 2:**
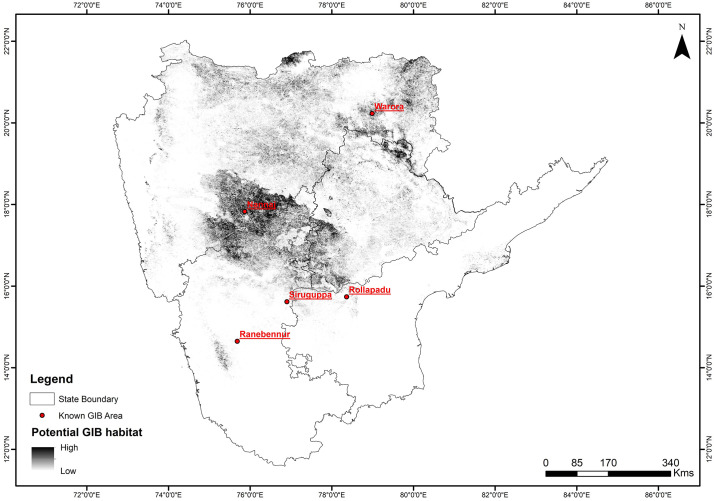
Potential probability distribution map of GIB in Landscape along with the known GIB areas in the landscape.

The GIB distribution map was predicted for over 7,20,000 km^2^ in the Deccan landscape covering the states of Maharashtra, Karnataka, Telangana, and Andhra Pradesh, India. We assigned suitable habitats for GIB based on equal test sensitivity and specificity logistic threshold [48]. We found that approximately 48,000 km^2^ (6.6%) of the total land area of the Deccan landscape is suitable for GIB (Fig 2). The potential suitable areas of Deccan landscapes were distributed in 35 districts (22 in Maharashtra, six in Karnataka and Telangana, and one in Andhra Pradesh) (S2 Table in S1 File).

The variables that contributed most to determining potential habitat suitability in Deccan Landscape were precipitation of warmest quarter Bio 18 (40.3%), mean diurnal range Bio 2 (12.7), NDVI of June (5.6%), NDVI of August month (5.2%), human settlement (5.2%), Annual Precipitation Bio 12 (4.7%), LULC (3.7%) distance from powerline (3.7%), human footprint (3.0%), Annual mean temperature (2.9%) and human population density (2.9%) influenced the GIB distribution (S1 Fig in S2 File). The average training and test AUC score of ten replicates for the model was 0.85 ± SD0.005 and 0.74 ± SD0.06, respectively. The TSS score was found to be 0.42 ± SD0.03 for the potential GIB distribution model.

### Statewise potential suitable habitat for GIB

The study revealed that suitable habitat for GIB comprised 7.49% of Maharashtra’s total area, 5.59% of Telangana, 4.48% of Karnataka, and 0.02% of Andhra Pradesh within the Deccan Landscape. In Maharashtra, Osmanabad and Solapur districts showed the highest suitable area of 66.97% and 50.91%, respectively. In Karnataka, the districts Gulbarga, Bijapur, and Bidar showed the most suitable areas, 31.04%, 28.97%, and 26.85%, respectively. The districts of Rangareddy, Mahbubnagar, and Adilabad in Telangana had the highest proportion of suitable habitat, covering 25.01%, 17.73%, and 15.71%, respectively. The result also showed suitable areas in known GIB locations, such as Kurnool, Nannaj Bustard Sanctuary, and Bidar districts. The result also predicted a potential area near the Siruguppa area of the Bellary District of Karnataka, a proposed site for the protection of GIB (S2 Table in S1 File).

### Identification of priority conservation areas using grid based survey

**Fig 3:**
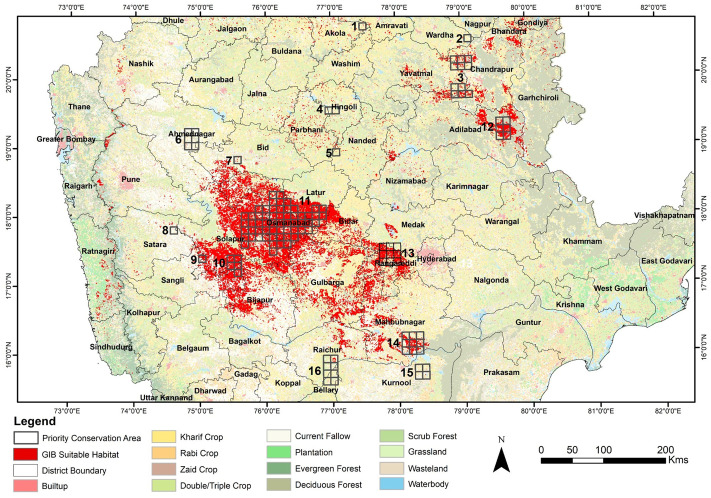
Priority conservation identified in different districts of Maharashtra. The detail of each critical site provided in the supplementary material (landuse landcover map source: LULC-AWiFS data for 2013−14 was obtained for the State of Maharashtra, Karnataka, Andhra Pradesh obtained vide request ID 14778−80 from Bhuvan Portal of NRSC.

A total of 2117 line transects covering a distance of 6436.6 km of mean transect length 3.03 ± 1.74 km (single continuous or multiple broken transects) in 372 grids in a slow-moving (10–20 km/hr) vehicle. During the survey, no GIB was sighted. However, through the questionnaire survey, 1401 respondents were interviewed. Out of these, 72 confirmed GIB presence in their area in the last six months. We combined this information with the species distribution model and identified 89 out of 372 grids as priority conservation areas for GIB in Maharashtra (Fig 3). These grids constituted 11 sites spread across 11 districts of Maharashtra, covering an area of 12,816 km^2^. We also identified five sites consisting of 36 grids of 12*12 km of the priority conservation area in Telangana, Karnataka, and Andhra Pradesh, covering an area of 5184 km^2^ based on the probability distribution map. Sixteen sites consisting of 125 grids were identified as the priority conservation area in the Deccan landscape. These identified grids were majorly dominated by fallow lands (3773 km^2^) and kharif crops (3731 km^2^), followed by grasslands (Table 1). We also evaluated the linear infrastructure and mining areas present within the priority conservation areas to understand the extent of pressure at each site (Fig 3; S2-S17 Figs in S2 File).

**Table 1:** Area-wise detail of landuse landcover composition (LULC-AWiFS data for the year 2013−14 from Bhuvan portal) and linear infrastructures (openstreet.org) of each identified site in the Deccan landscape.

Feature	Cluster 1	Cluster 2	Cluster 3	Cluster 4	Cluster 5	Cluster 6	Cluster 7	Cluster 8	Cluster 9	Cluster 10	Cluster 11	Cluster 12	Cluster 13	Cluster 14	Cluster 15	Cluster 16
Total area (km^2^)	144	144	1584	288	144	864	144	144	144	720	8496	864	1296	1296	576	1152
**Land Use Land Cover Feature (%)**																
Builtup	1.5	0.5	2.5	1.1	2.1	2.2	0.6	1.0	1.2	0.5	2.0	2.2	3.3	3.6	2.2	2.0
Kharif Crop	56.7	45.2	44.3	30.1	62.0	13.6	25.6	3.7	2.5	4.9	20.3	32.6	35.4	26.0	12.1	24.6
Rabi Crop	2.3	1.4	0.9	6.4	0.0	5.0	2.5	4.1	8.4	5.8	8.0	0.4	1.7	11.2	30.2	10.1
Zaid Crop	0.0	0.0	0.0	0.4	0.0	0.0	0.0	0.0	0.1	0.3	0.3	0.0	0.0	0.0	0.0	0.00
Mixed Crop	16.7	12.8	18.8	20.8	30.4	3.7	5.3	1.2	1.6	2.6	20.4	4.6	37.7	6.4	13.4	39.4
Current Fallow	17.4	14.1	12.8	14.9	0.5	50.7	30.1	52.8	70.4	72.6	32.6	20.1	7.8	23.9	21.5	16.2
Plantation	0.0	0.1	0.0	0.3	0.0	0.1	0.2	0.0	0.0	0.1	0.2	0.9	0.0	1.2	0.0	0.3
Decidous Forest	1.3	11.2	6.7	6.5	0.0	2.8	1.8	0.1	0.0	0.1	0.2	20.9	2.2	2.4	0.0	0.0
Degraded Forest	0.1	1.4	3.1	1.9	0.0	3.8	6.1	1.4	0.0	0.1	0.1	4.0	0.9	1.2	0.0	0.2
Wasteland	1.8	11.1	8.9	11.4	3.3	17.0	26.0	34.1	13.1	11.4	12.5	8.6	8.5	14.0	17.2	2.9
Water	2.1	2.4	2.2	6.2	1.6	1.1	1.7	1.5	2.7	1.6	3.3	5.7	2.5	10.2	3.3	4.3
Mines	0.0	0.0	0.0	0.0	0.0	0.0	0.0	0.0	0.0	0.0	1.7	0.0	0.0	0.0	0.0	0.0
**Linear Infrastructure Feature (km)**																
Railway track	40.0	0.0	52.8	0.0	0.0	242.0	0.0	0.0	0.0	0.0	64.6	0.0	95.0	0.0	0.0	29.0
Roads	73.8	0.0	330.1	63.4	43.5	382.7	50.3	7.7	35.1	367.6	1944.1	0.0	129.4	119.0	16.7	204.6
Powerline	242.1	401.1	2349.1	86.3	0.0	181.1	332.3	0.0	0.0	219.7	1687.5	37.7	64.2	159.4	104.7	132.0
Canal	0.0	0.0	89.7	13.0	39.9	0.0	0.0	32.4	7.5	236.3	647.7	52.8	13.4	188.4	55.8	52.3

### Comparison between priority conservation areas and random sites

**Fig 4:**
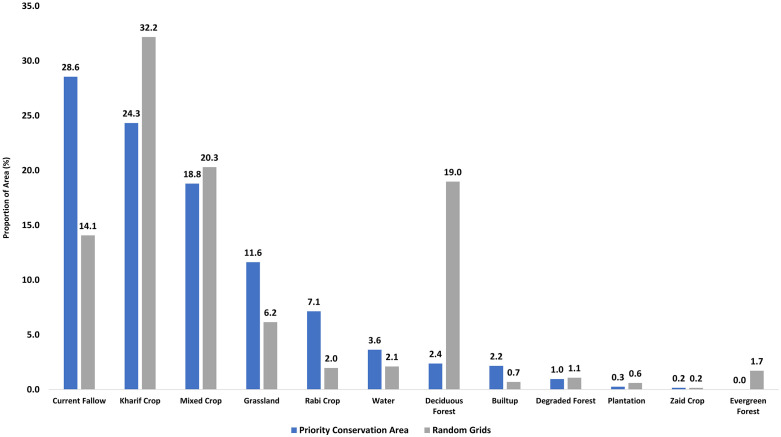
Difference in land use land cover classes between the identified priority conservation areas and randomly selected grids (n = 125 grids of 12*12 km). LULC-AWiFS data was obtained for the State of Maharashtra, vide request ID 14778 from Bhuvan Portal of NRSC.

The comparison of land use and land cover (LULC) between priority conservation areas and randomly selected sites revealed significant differences in several categories (Fig 4). Current fallow land accounted for a significantly higher proportion in priority conservation areas (28.6%) compared to random sites (14.1%) (p = 0.01). Similarly, Grasslands also covered a larger proportion in conservation areas (11.6%) than in random sites (6.2%). Kharif cropland was significantly less dominant in priority conservation areas (24.3%) than in random sites (32.2%) (p = 0.01).

On the other hand, deciduous forest was significantly more abundant in random sites (19.0%) than in priority conservation areas (2.4%) (p = 0.001), suggesting a lower forest cover in conservation priority zones. Rabi cropland, water bodies, and built-up areas were also more prevalent in priority conservation areas than in random sites. Other land cover types, such as degraded forests, plantations, and zaid crops, had minimal representation in both site categories, with only slight variations (Fig 4).

## Discussion

The study indicated that only 6.6% of the total area from four states was suitable for GIB in the landscape. The results showed that precipitation is one of the important variables in predicting GIB distribution, as they preferred a precise range of precipitation. The highest contributing variable was the precipitation of the warmest month, which is fall in April to May in the landscape, and the mean diurnal range is explained in terms of the preference for arid and semi-arid habitats by the GIB. The warmest month coincides with the monsoon season (June to September), providing essential water and promoting the growth of grasses and insects, which are vital for the bird’s diet and breeding success. Moderate precipitation during the warmest quarter in the dry landscape ensures suitable foraging grounds and nesting sites, thus significantly contributing to the habitat suitability for the species (S1 Fig in S2 File). Ecological studies of GIB also report its presence in areas with relatively low to moderate rainfall (~600 mm) in the Deccan landscape [57,58]. Additionally, theoretical and empirical ecological research on semi-arid ecosystems suggests that both insufficient and very high precipitation regimes can negatively affect vegetation structure and water availability [59].

Furthermore, the NDVI of June and August is crucial for identifying suitable habitats for the GIB. These months represent the onset of the monsoon, which enhances vegetation growth, providing the necessary cover and food resources like grasses and insects that are vital for the birds’ foraging and breeding. High NDVI values during these months indicate lush vegetation, which is essential for nesting and protection from predators. Thus, NDVI data from June and August are significant indicators of habitat suitability for the species, reflecting the seasonal vegetation dynamics critical for its survival. The response curve indicates that GIB prefers moderate vegetation greenness during June-August, typical of semi-arid grasslands after early monsoon rainfall. Very high NDVI, associated with dense vegetation or agricultural growth, sharply reduces suitability, as the species avoids tall or dense vegetation and human-dominated croplands. Extremely low NDVI also reduces suitability due to a lack of forage and nesting cover (S1 Fig in S2 File). Our results suggested that the rabi crop, fallow lands, wastelands (or grasslands), plantations, and water availability are significant for their distribution (S1 Fig in S2 File). With the decreasing habitat and population, it is necessary to identify critical areas for GIB survival.

Our model identified a potential habitat for GIB in and around the Rollapadu Wildlife Sanctuary in Andhra Pradesh, one of the country’s smallest wildlife sanctuaries, and recorded three females in 2019 [26]. However, they were not spotted in the recent survey conducted in 2024 in the sanctuary and its surroundings [60]. Moreover, the Nannaj bustard sanctuary also recorded a single female who visited the sanctuary during the breeding season [61]. Later, in 2020, 2021, 2022, 2023, and 2024, the female bird was again sighted by locals near the sanctuary. These two PAs have records of GIB, but all the recorded individuals were females. The sanctuaries require males to breed with the females so that the population can sustain itself in the landscape. The only other sanctuary in Southern India where bustards were found was the Ranebennur Blackbuck Sanctuary in Karnataka. According to an official report of the Karnataka Forest Department, no bird has been recorded since 1988. The status of the bird in Karnataka state is on the verge of extinction. For this, the Karnataka Forest Department is targeting an area of 485.62 hectares in the Siruguppa area of the Bellary district to conserve GIB, where there is a record of 3–4 birds [56,62]. These patchy distributions and biased sex ratios are alarming and make immediate conservation measures necessary to save the birds in the landscape. Most of these sanctuaries have either been denotified or reduced in size over the years due to various administrative and land-use pressures. GIBs are wide-ranging birds that require a mosaic of habitats to meet their life-history needs. During the breeding season, males depend on open, undisturbed areas for displaying, mating, and nesting, making such habitats critical for the species’ long-term survival. Recent sightings, although few, highlight the importance of safeguarding these landscapes. Protecting and maintaining these areas in their natural state is essential, as they hold potential for future GIB recovery efforts and reintroduction programmes.

We have identified priority conservation areas from different regions of the Deccan landscape, ranging from 144–8496 km^2^ at 16 sites by combining SDM and questionnaire survey. These sites were dominated by kharif crops, followed by open lands (fallow lands and grasslands). We have also evaluated the anthropogenic pressures in the identified conservation areas, such as road and railway networks, powerlines, and legal mining areas. This information will help understand the level of effort and management intervention required for each site to ensure GIB’s existence in the region. Detailed information on each site’s land-use types, powerlines, railway, road, and canal networks is provided in the supplementary material (S2-S17 Figs in S2 File).

The identified priority conservation areas were dominated by fallow lands (28.6%), kharif crops (24.3%), mixed crops (18.8%), and grasslands (11.6%). Compared with 125 randomly selected grids, fallow lands and kharif crops were significantly higher in priority areas (p = 0.01; Fig 4). These land-use types are ecologically important for GIB conservation due to their strong behavioural relevance [44]. Kharif croplands provide abundant food resources, such as insects, seeds, and plant matter, during the monsoon, making them key foraging habitats [24]. Adjacent grasslands offer suitable nesting sites and open spaces essential for courtship displays, breeding, and chick rearing [63]. Together, this mosaic of croplands and grasslands supports multiple life-history requirements of the species.

Our study demonstrates an effective approach for identifying priority conservation areas for species persisting largely in human-dominated landscapes, thereby supporting conservation planning beyond the protected area network. Using landscape-level surveys and distribution modelling, we identified key areas for GIB conservation across Maharashtra, Karnataka, Andhra Pradesh, and Telangana. The modelling showed that GIB presence is strongly influenced by temperature, precipitation, vegetation cover, and human population density, with higher habitat suitability in regions practicing traditional cropping systems, such as Solapur and Osmanabad. In these areas, farmers typically maintain a mix of cultivated and fallow lands, either due to climatic constraints, crop loss, or low economic returns, which creates a mosaic of habitats that GIBs extensively use [23,64].

However, over the past century, India has experienced substantial loss of grasslands and other open habitats due to agricultural expansion, infrastructure growth, and rapid shifts from traditional food-crop systems to cash crops like sugarcane and cotton [27,28,65]. This widespread transition across the dryland regions of Maharashtra, Karnataka, Telangana, and Andhra Pradesh has reduced the availability of fallow lands and altered the open landscapes critical for GIB survival. The resulting habitat changes have had major ecological consequences for species dependent on these open ecosystems, underscoring the urgency of conserving remnant traditional agricultural landscapes for the GIB.

To save the species from extinction, the Ministry of Environment, Forest and Climate Change (MoEFCC), the Rajasthan Government, and the Wildlife Institute of India (WII) jointly started a captive breeding facility for GIB in Rajasthan, India. Since 2019, experts have collected eggs from the wild, successfully incubated them, and raised a breeding adult population in captivity. The goal is to build a secure captive stock and eventually release future generations into the wild to restore viable populations.

In parallel with these ex-situ efforts, it is critical to identify and protect suitable habitats for future translocation and reintroduction. The priority conservation areas identified in the Deccan landscape are especially valuable, offering potential release sites that can support long-term survival. These landscapes face multiple threats, particularly powerlines, habitat loss, and other anthropogenic disturbances, making targeted conservation essential. Safeguarding these areas will benefit not only the GIB by enabling population recovery and genetic resilience but also broader regional biodiversity. Additionally, well-managed GIB habitats can provide sustainable livelihood opportunities for local communities and help preserve the ecological and cultural heritage of the region.

This study demonstrates how habitat suitability models, combined with telemetry data, offer a robust framework for identifying priority conservation areas for the GIB. The Deccan landscape, home to some of the last remaining GIB individuals in southern India, emerges as a key region with high conservation potential. Although the population in this landscape has declined sharply, the habitat itself remains relatively less degraded compared to other parts of the species’ range, making it a strong candidate for future reintroduction and population recovery efforts. Promoting traditional, low-intensity land-use practices can further enhance habitat suitability and support the species’ ecological requirements.

Importantly, this finding aligns with the broader argument made by Madhusudan and Vanak [66] that India’s Open Natural Ecosystems (ONE), including grasslands, savannas, fallows, and scrublands, are among the most overlooked yet biodiversity-rich ecosystems in the country. These ecosystems support a wide array of specialized and threatened species, including bustards, floricans, wolves, and blackbuck. Their work emphasizes that misclassification of ONE as “wastelands” has accelerated widespread conversion, undermining the ecological integrity of landscapes crucial for species that depend on open habitats. In this context, our integrated approach using species distribution modelling and GPS telemetry helps identify precisely those ONE landscapes that remain functionally intact and can serve as critical insurance sites for securing the long-term survival of the GIB outside traditional protected areas. Conserving these open ecosystems is therefore essential not only for the GIB but also for safeguarding many other species uniquely adapted to India’s natural open habitats.

Beyond its direct relevance to GIB conservation, this study provides a replicable framework for species facing similar threats in human-dominated landscapes globally. Our spatial ecology-based approach, which integrates habitat suitability modelling with landscape-level assessments, can be adapted for other taxa where conventional protected areas may be insufficient. By identifying high-priority conservation zones and aligning them with ecological and socio-economic contexts, this method supports more targeted, effective, and scalable conservation planning. The study also underscores the need for proactive measures to prevent local extirpation, restore populations across their former ranges, and enhance connectivity among fragmented habitats to reduce risks such as inbreeding depression and genetic bottlenecks. Overall, the insights and tools presented here offer a valuable pathway for safeguarding endangered species worldwide under increasing anthropogenic pressures.

## Supporting information

S1 File Contains two tables, list of all environmental variables acquired along with descriptions for conducting species distribution modeling of Great Indian Bustard in Deccan Landscape (S1 Table) and suitable habitat area-wise details of 35 districts (with more than 1% of suitable habitat of total area) of four states (Maharashtra, Karnataka, Andhra Pradesh, and Telangana) of Deccan Landscape (S2 Table). (DOCX)

S2 File Containes supplementary figures illustrating species distribution model response curves (S1 Fig) and priority conservation areas identified during landscape surveys in western and peninsular India (S2-S17 Figs). (DOCX)

## References

[1] Khoshoo TN. Census of India’s biodiversity: tasks ahead. Curr Sci. 1995;69(14):14–7.

[2] Myers N, Mittermeier RA, Mittermeier CG, da Fonseca GA, Kent J. Biodiversity hotspots for conservation priorities. Nature. 2000;403(6772):853–8. doi: 10.1038/35002501 10706275

[3] Morrison JC, Sechrest W, Dinerstein E, Wilcove DS, Lamoreux JF. Persistence of large mammal faunas as indicators of global human impacts. J Mammal. 2007;88(6):1363–80. doi: 10.1644/06-mamm-a-124r2.1

[4] Velho N, Karanth KK, Laurance WF. Hunting: a serious and understudied threat in India, a globally significant conservation region. Biol Conserv. 2012;148:210–5.

[5] Tandon P, Abrol YP, Kumaria S. Biodiversity and its significance. New Delhi, India: I. K. International Publishing House Pvt. Ltd.; 2007.

[6] Mace GM, Collar NJ, Gaston KJ, Hilton-Taylor C, Akçakaya HR, Leader-Williams N, et al. Quantification of extinction risk: IUCN’s system for classifying threatened species. Conserv Biol. 2008;22(6):1424–42. doi: 10.1111/j.1523-1739.2008.01044.x 18847444

[7] Wilder AP, Supple MA, Subramanian A, Mudide A, Swofford R, Serres-Armero A, et al. The contribution of historical processes to contemporary extinction risk in placental mammals. Science. 2023;380(6643):eabn5856. doi: 10.1126/science.abn5856 37104572 PMC10184782

[8] Perzanowski K, Bleyhl B, Olech W, Kuemmerle T. Connectivity or isolation? Identifying reintroduction sites for multiple conservation objectives for wisents in Poland. Anim Conserv. 2019;23:212–21.

[9] Hannah L, Midgley G, Andelman S, Araújo M, Hughes G, Martinez-meyer E, et al. Protected area needs in a changing climate. Front Ecol Environ. 2007;5:131–8.

[10] Viña A, Liu J. Hidden roles of protected areas in the conservation of biodiversity and ecosystem services. Ecosphere. 2017;8(6). doi: 10.1002/ecs2.1864

[11] Jiang G, Wang G, Holyoak M, Yu Q, Jia X, Guan Y, et al. Land sharing and land sparing reveal social and ecological synergy in big cat conservation. Biol Conserv. 2017;211:142–9.

[12] Teixeira CP, de Azevedo CS, Mendl M, Cipreste CF, Young RJ. Revisiting translocation and reintroduction programmes: the importance of considering stress. Anim Behav. 2007;73:1–13.

[13] Kuemmerle T, Perzanowski K, Akçakaya HR, Beaudry F, Van Deelen TR, Parnikoza I, et al. Cost-effectiveness of strategies to establish a European bison metapopulation in the Carpathians. J Appl Ecol. 2011;48:317–29.

[14] Jayadevan A, Nayak R, Karanth KK, Krishnaswamy J, DeFries R, Karanth KU, et al. Navigating paved paradise: evaluating landscape permeability to movement for large mammals in two conservation priority landscapes in India. Biol Conserv. 2020;247:108613.

[15] Rahmani AR, Manakadan R. The past and present distribution of the Great Indian Bustard *Ardeotis nigriceps* (Vigors) in India. J Bombay Nat History Soc. 1990;87(2).

[16] Islam MZ, Rahmani AR. Threatened Birds of India. Buceros: Oxford University Press BNHS and BirdLife International (UK); 2002.

[17] Dutta S, Jhala Y. Devil is in the detail: behaviorally explicit habitat selection by the Critically Endangered great Indian bustard. Endang Species Res. 2021;45:55–69. doi: 10.3354/esr01126

[18] Khan S, Chatterjee N, Habib B. Testing performance of large-scale surveys in determining trends for the critically endangered Great Indian Bustard Ardeotis nigriceps. Sci Rep. 2019;9(1):11627. doi: 10.1038/s41598-019-48193-2 31406220 PMC6690994

[19] Jhala YV, Dutta S, Karkarya T, Awasthi A, Bipin CM, Anoop KR, et al. Habitat improvement and conservation breeding of the Great Indian Bustard: An Integrated Approach. Dehradun, India: Wildlife Institute of India; 2020. pp. 1–134.

[20] Birdlife International. Great Indian Bustard (*Ardeotis nigriceps*) BirdLife species factsheet. 2020. Available from: https://datazone.birdlife.org/species/great-indian-bustard-ardeotis-nigriceps

[21] Rao KT, Javed SMM. The Great Indian Bustard *Ardeotis nigriceps* (Vigors) in and around the Rollapadu Wildlife Sanctuary, Andhra Pradesh, India. Zoos Print J. 2005;20(11):2053–8. doi: 10.11609/jott.zpj.1326.2053-8

[22] Thosar G, Ladkhedkar R, Pimplapure A, Kasambe R. Status and conservation of Great Indian Bustards in Vidarbha. Mistnet. 2007;22:201–4.

[23] Habib B, Khan S, Talukdar G, Kumar RS. Status of Great Indian Bustard and associated species in the State of Maharashtra, India - 2017. Dehradun: Wildlife Institute of India. 2018.

[24] Rahmani AR. Need to start project bustards. Mumbai: Bombay Natural History Society; 2006.

[25] Dutta S, Rahmani AR, Jhala YV. Running out of time? The great Indian bustard Ardeotis nigriceps: status, viability, and conservation strategies. Eur J Wildl Res. 2010;57:615–25.

[26] Vidura T. Fall of the Great Indian Bustard. The Hindu. Andhra Pradesh. 2019. [cited 2020 Apr 18]. Available from: https://www.thehindu.com/news/national/andhra-pradesh/fall-of-the-great-indian-bustard/article30126253.ece

[27] Tian H, Banger K, Bo T, Dadhwal VK. History of land use in India during 1880–2010: Large-scale land transformations reconstructed from satellite data and historical archives. Glob Planet Change. 2014;121:78–88. doi: 10.1016/j.gloplacha.2014.07.005

[28] Nalawade DB, Chavan SM, Pawar CT. Spatio temporal changes in cropping pattern of South Konkan of Maharashtra: A Geographical Analysis, Cyber Literature. Int Online J. 2010;3:76–83.

[29] Seethalakshmi P. Shifting land use pattern in Andhra Pradesh: Implications for agricultural and food security. Hyderabad, Andhra Pradesh: Center for Sustainable Agriculture; 2010.

[30] Janakiraman J, Jalal JS. Angiosperm diversity of the Great Indian Bustard Wildlife Sanctuary: A semi-arid grassland, Maharashtra, India. Check List. 2015;11(2):1–20.

[31] Ishtiaq F, Dutta S, Yumnam B, Jhala YV. Low genetic diversity in the endangered Indian bustard (*Ardeotis nigriceps*) across India and implications for conservation. Conserv Genet. 2011;12:857–63.

[32] Guisan A, Broennimann O, Engler R, Vust M, Yoccoz NG, Lehmann A, et al. Using niche-based models to improve the sampling of rare species. Conserv Biol. 2006;20(2):501–11. doi: 10.1111/j.1523-1739.2006.00354.x 16903111

[33] Franklin J. Species distribution models in conservation biogeography: developments and challenges. Divers Distrib. 2013;19(10):1217–23. doi: 10.1111/ddi.12125

[34] Eyre A, Briscoe N, Harley D, Lumsden L, McComb L, Lentini P. Using species distribution models and decision tools to direct surveys and identify potential translocation sites for a critically endangered species. Divers Distrib. 2022;28:700–11. doi: 10.1111/ddi.13469

[35] Dayton GH, Fitzgerald LA. Habitat suitability models for desert amphibians. Biol Conserv. 2006;132(1):40–9. doi: 10.1016/j.biocon.2006.03.012

[36] Wisz M, Dendoncker N, Madsen J, Rounsevell M, Jespersen M, Kuijken E, et al. Modelling pink-footed goose (Anser brachyrhynchus) wintering distributions for the year 2050: potential effects of land-use change in Europe. Divers Distrib. 2008;14:721–31.

[37] Edrén SMC, Wisz MS, Teilmann J, Dietz R, Söderkvist J. Modelling spatial patterns in harbour porpoise satellite telemetry data using maximum entropy. Ecography. 2010;33(4):698–708. doi: 10.1111/j.1600-0587.2009.05901.x

[38] Sofaer HR, Jarnevich CS, Pearse IS, Smyth RL, Auer S, Cook GL, et al. Development and delivery of species distribution models to inform decision-making. BioScience. 2019;69(7):544–57. doi: 10.1093/biosci/biz045

[39] Wilting A, Cord A, Hearn AJ, Hesse D, Mohamed A, Traeholdt C, et al. Modelling the species distribution of flat-headed cats (Prionailurus planiceps), an endangered South-East Asian small felid. PLoS One. 2010;5(3):e9612. doi: 10.1371/journal.pone.0009612 20305809 PMC2840020

[40] Clements GR, Rayan DM, Aziz SA, Kawanishi K, Traeholt C, Magintan D, et al. Predicting the distribution of the Asian tapir in Peninsular Malaysia using maximum entropy modeling. Integr Zool. 2012;7(4):400–6. doi: 10.1111/j.1749-4877.2012.00314.x 23253371

[41] Vale CG, Campos JC, Silva TL, Gonçalves DV, Sow AS, Martínez-Freiría F, et al. Biogeography and conservation of mammals from the west Sahara-sahel: an application of ecological niche-based models and GIS. Hystrix. 2016;27:1–10.

[42] Coxen CL, Frey JK, Carleton SA, Collins DP. Species distribution models for a migratory bird based on citizen science and satellite tracking data. Glob Ecol Conserv. 2017;11:298–311.

[43] Lundy MG, Buckley DJ, Boston ESM, Scott DD, Prodöhl PA, Marnell F, et al. Behavioural context of multi-scale species distribution models assessed by radio-tracking. Bas Appl Ecol. 2012;13:188–95.

[44] Habib B, Talukdar G, Kumar RS, Limaye S, Vaijayanti V. Tracking the Great Indian Bustard in Maharashtra India. Dehradun: Wildlife Institute of India; 2016.

[45] Dutta S, Bhardwaj GS, Bhardwaj DK, Jhala YV. Status of Great Indian Bustard and Associated Wildlife in Thar. Dehradun: Wildlife Institute of India, Rajasthan Forest Department, Jaipur. 2014.

[46] Phillips SJ, Anderson RP, Schapire RE. Maximum entropy modeling of species geographic distributions. Ecol Modell. 2006;190(3–4):231–59. doi: 10.1016/j.ecolmodel.2005.03.026

[47] Elith J, Phillips SJ, Hastie T, Dudi’k M, Chee YE, Yates CJ. A statistical explanation of MaxEnt for ecologists. Divers Distrib. 2011;17:43–57.

[48] Fick SE, Hijmans RJ. Worldclim 2: New 1-km spatial resolution climate surfaces for global land areas. Int J Climatol. 2017;37(12):4302–15.

[49] WorldPop. Global high-resolution population denominators project. The Bill and Melinda Gates Foundation; 2018.

[50] Dudík M, Schapire RE, Phillips SJ. Correcting sample selection bias in maximum entropy density estimation. Adv Neural Inf Process Syst. 2005;18:323–30.

[51] Phillips SJ, Dudík M, Elith J, Graham CH, Lehmann A, Leathwick J, et al. Sample selection bias and presence-only distribution models: implications for background and pseudo-absence data. Ecol Appl. 2009;19(1):181–97. doi: 10.1890/07-2153.1 19323182

[52] Fourcade Y, Engler JO, Rödder D, Secondi J. Mapping species distributions with MAXENT using a geographically biased sample of presence data: a performance assessment of methods for correcting sampling bias. PLoS One. 2014;9(5):e97122. doi: 10.1371/journal.pone.0097122 24818607 PMC4018261

[53] Allouche O, Tsoar A, Kadmon R. Assessing the accuracy of species distribution models: prevalence, kappa and the true skill statistic (TSS). J Appl Ecol. 2006;43:1223–32.

[54] Meng J, Li M, Guo J, Zhao D, Tao J. Predicting Suitable Environments and Potential Occurrences for Cinnamomum camphora (Linn.) Presl. Forests. 2021;12(8):1126. doi: 10.3390/f12081126

[55] Roman J, Ehrlich P, Pringlex J, Avise J. Facing Extinction: Nine Steps to Save Biodiversity. Solution. 2009;1(1):50–61.

[56] Kumar PR, Deepak D, Kumara HN, Babu S. The occupancy of Great Indian Bustard (*Ardeotis nigriceps*) using local people’s knowledge in the Deccan Plateau, Karnataka, India. Biodiversitas. 2023;24(3). doi: 10.13057/biodiv/d240309

[57] Munjpara SB, Pandey CN, Jethva B. Habitat use by the Great Indian Bustard Ardeotis nigriceps (Gruiformes: Otididae) in breeding and non-breeding seasons in Kachchh, Gujarat, India. JoTT. 2012;5(2):3654–60. doi: 10.11609/JoTT.o2757.3654-60

[58] Khan S, Vijayaraghavan V, Talukdar G, Kumar RS, Habib B. Not all those who wander are lost: Insights into movement pattern of the great Indian bustard in the Deccan landscape of India. Ecol Evol. 2025;15(7):e71742. doi: 10.1002/ece3.71742 PMC12230192 40625326

[59] Ottó B, Végvári Z. Bioclimatic Preferences of the Great Bustard in a Steppe Region. Diversity. 2022;14(12):1138. doi: 10.3390/d14121138

[60] Pulipaka B. No more Great Indian Bustard in Andhra Pradesh. Deccan Cronicles. Hyderabad, Andhra Pradesh. 2022. [cited 2023 Jan 15]. https://www.deccanchronicle.com/nation/in-other-news/020522/no-more-great-indian-bustard-in-ap-on-their-way-out-in-south-india.html

[61] Nair M. Just one sighting of Great Indian Bustard last year worries foresters. The Times of India. Pune. 2019. [cited 2023 Jan 15]. https://timesofindia.indiatimes.com/city/pune/just-one-sighting-of-great-indian-bustard-last-year-worries-foresters/articleshow/68786308.cms

[62] Bhardwaj M. Siruguppa may become Great Indian Bustard protection zone. New Indian Express Ballari. 2018. [cited 2023 Jan 15]. https://www.newindianexpress.com/states/karnataka/2018/Dec/25/siruguppa-may-become-great-indian-bustard-protection-zone-1916257.html

[63] Rahmani A. Protection for the great Indian bustard. Oryx. 1987;21(3):174–9. doi: 10.1017/s0030605300026922

[64] Sharma TC. Technological change in Indian agriculture. A regional perspective. Jaipur and New Delhi: Rawat Publications; 1999.

[65] Banerjee S, Das D, Zhang H, John R. Grassland-woodland transitions over decadal timescales in the Terai-Duar savanna and grasslands of the Indian subcontinent. For Ecol Manag. 2023;530:120764.

[66] Madhusudan MD, Vanak AT. Mapping the distribution and extent of India’s semi‐arid open natural ecosystems. J Biogeogr. 2023;50(8):1377–87.

