# Supplementary material for: In Search of Safe Havens: Identifying priority conservation areas for a critically endangered bird, the Great Indian Bustard, in the Deccan Landscape of India
Source: PLoS One. 2026 Aug 14;21(8):e0355780. doi: 10.1371/journal.pone.0355780 (PMC13475822; doi:10.1371/journal.pone.0355780)
Supplement: Supplementary file 1 [file pone.0355780.s001.docx]

**Appendix 1**

**S1 Table. List of all environmental variables acquired along with descriptions for conducting species distribution modeling of Great Indian Bustard in Deccan Landscape.**

| S.No. | Variable | Description | Source |
| --- | --- | --- | --- |
| 1 | *Annual Mean Temperature (Bio 1) | Continuous | http://www.worldclim.org/bioclim |
| 2 | *Mean Diurnal Range (Bio 2) | Continuous |  |
| 3 | Isothermality (Bio3) | Continuous |  |
| 4 | Temperature Seasonality (Bio 4) | Continuous |  |
| 5 | Max Temperature of Warmest Month (Bio 5) | Continuous |  |
| 6 | Min Temperature of Coldest Month (Bio 6) | Continuous |  |
| 7 | Temperature Annual Range (Bio 7) | Continuous |  |
| 8 | Mean Temperature of Wettest Quarter (Bio 8) | Continuous |  |
| 9 | Mean Temperature of Driest Quarter (Bio 9) | Continuous |  |
| 10 | Mean Temperature of Warmest Quarter (Bio 10) | Continuous |  |
| 11 | Mean Temperature of Coldest Quarter (Bio 11) | Continuous |  |
| 12 | *Annual Precipitation (Bio 12) | Continuous |  |
| 13 | Precipitation of Wettest Month (Bio 13) | Continuous |  |
| 14 | *Precipitation of Driest Month (Bio 14) | Continuous |  |
| 15 | Precipitation Seasonality (Bio 15) | Continuous |  |
| 16 | Precipitation of Wettest Quarter (Bio 16) | Continuous |  |
| 17 | Precipitation of Driest Quarter (Bio 17) | Continuous |  |
| 18 | *Precipitation of Warmest Quarter (Bio 18) | Continuous |  |
| 19 | *Precipitation of Coldest Quarter (Bio 19) | Continuous |  |
| 20 | *mod13q1.a2013001_16_days_ndvi | Continuous | https://modis.gsfc.nasa.gov/data |
| 21 | mod13q1.a2013017_16_days_ndvi | Continuous |  |
| 22 | mod13q1.a2013033_16_days_ndvi | Continuous |  |
| 23 | mod13q1.a2013049_16_days_ndvi | Continuous |  |
| 24 | mod13q1.a2013065_16_days_ndvi | Continuous |  |
| 25 | mod13q1.a2013097_16_days_ndvi | Continuous |  |
| 26 | mod13q1.a2013113_16_days_ndvi | Continuous |  |
| 27 | mod13q1.a2013129_16_days_ndvi | Continuous |  |
| 28 | mod13q1.a2013145_16_days_ndvi | Continuous |  |
| 29 | mod13q1.a2013161_16_days_ndvi | Continuous |  |
| 30 | *mod13q1.a2013177_16_days_ndvi | Continuous |  |
| 31 | *mod13q1.a2013193_16_days_ndvi | Continuous |  |
| 32 | *mod13q1.a2013209_16_days_ndvi | Continuous |  |
| 33 | *mod13q1.a2013225_16_days_ndvi | Continuous |  |
| 34 | *mod13q1.a2013241_16_days_ndvi | Continuous |  |
| 35 | mod13q1.a2013257_16_days_ndvi | Continuous |  |
| 36 | mod13q1.a2013273_16_days_ndvi | Continuous |  |
| 37 | *mod13q1.a2013289_16_days_ndvi | Continuous |  |
| 38 | mod13q1.a2013305_16_days_ndvi | Continuous |  |
| 39 | mod13q1.a2013321_16_days_ndvi | Continuous |  |
| 40 | mod13q1.a2013337_16_days_ndvi | Continuous |  |
| 41 | mod13q1.a2013353_16_days_ndvi | Continuous |  |
| 42 | *Distance to Powerline | Continuous | https://www.openstreetmap.org/export#map |
| 43 | *Bhuvan Land Use Land Cover | Categorical | https://bhuvan-app1.nrsc.gov.in/thematic/thematic/index.php |
| 44 | *Human Footprint | Continuous | https://sedac.ciesin.columbia.edu/ |
| 45 | *Global Human Settlement | Categorical | http://data.europa.eu/89h/jrc-ghsl-ghs_smod_pop_globe_r2016a |
| 46 | *Population Density 2015 | Continuous | https://www.worldpop.org/geodata/listing?id=64 |

***Note: Variables with (asterisks *) used for final modeling after conducting Pearson correlation test ( < 0.6 removed from analysis)***

**S2 Table. Suitable habitat area-wise details of 35 districts (with more than 1% of suitable habitat of total area) of four states (Maharashtra, Karnataka, Andhra Pradesh, and Telangana) of Deccan Landscape**

| S.no. | State | District | Total Area of District (km^2^) | Suitable Habitat (km^2^) | % Suitable Habitat |
| --- | --- | --- | --- | --- | --- |
| 1 | Maharashtra | Akola | 5523 | 624 | 11.31 |
| 2 | Maharashtra | Amravati | 12106 | 1747 | 14.43 |
| 3 | Maharashtra | Bhandara | 4693 | 944 | 20.10 |
| 4 | Maharashtra | Beed | 11402 | 721 | 6.32 |
| 5 | Maharashtra | Buldana | 9597 | 326 | 3.40 |
| 6 | Maharashtra | Chandrapur | 11366 | 1852 | 16.29 |
| 7 | Maharashtra | Dhule | 7142 | 288 | 4.03 |
| 8 | Maharashtra | Garhchiroli | 14515 | 803 | 5.53 |
| 9 | Maharashtra | Gondiya | 4657 | 1043 | 22.40 |
| 10 | Maharashtra | Hingoli | 4688 | 261 | 5.56 |
| 11 | Maharashtra | Jalgaon | 11714 | 797 | 6.80 |
| 12 | Maharashtra | Jalna | 7769 | 203 | 2.61 |
| 13 | Maharashtra | Latur | 7236 | 1879 | 25.97 |
| 14 | Maharashtra | Nanded | 10596 | 450 | 4.24 |
| 15 | Maharashtra | Nashik | 15478 | 168 | 1.08 |
| 16 | Maharashtra | Osmanabad | 7542 | 5051 | 66.97 |
| 17 | Maharashtra | Parbhani | 6328 | 749 | 11.84 |
| 18 | Maharashtra | Sangli | 8527 | 1253 | 14.69 |
| 19 | Maharashtra | Solapur | 14867 | 7569 | 50.91 |
| 20 | Maharashtra | Wardha | 6326 | 126 | 1.99 |
| 21 | Maharashtra | Washim | 5189 | 146 | 2.82 |
| 22 | Maharashtra | Yavatmal | 13559 | 896 | 6.61 |
| 23 | Andhra Pradesh | Kurnool | 17815 | 193 | 1.08 |
| 24 | Karnataka | Bagalkot | 6549 | 73 | 1.12 |
| 25 | Karnataka | Bidar | 5432 | 1459 | 26.85 |
| 26 | Karnataka | Bijapur | 10476 | 3035 | 28.97 |
| 27 | Karnataka | Gulbarga | 16222 | 5036 | 31.04 |
| 28 | Karnataka | Raichur | 8437 | 617 | 7.31 |
| 29 | Karnataka | Shimoga | 7733 | 251 | 3.24 |
| 30 | Telangana | Adilabad | 16101 | 2529 | 15.71 |
| 31 | Telangana | Karimnagar | 11954 | 137 | 1.14 |
| 32 | Telangana | Mahbubnagar | 18400 | 3262 | 17.73 |
| 33 | Telangana | Medak | 9776 | 573 | 5.86 |
| 34 | Telangana | Nizamabad | 7923 | 365 | 4.61 |
| 35 | Telangana | Rangareddi | 7564 | 1892 | 25.01 |
