# Supplementary material for: In Search of Safe Havens: Identifying priority conservation areas for a critically endangered bird, the Great Indian Bustard, in the Deccan Landscape of India
Source: PLoS One. 2026 Aug 14;21(8):e0355780. doi: 10.1371/journal.pone.0355780 (PMC13475822; doi:10.1371/journal.pone.0355780)
Supplement: Supplementary file 2 [file pone.0355780.s002.docx]

**Figures**

**S1 Fig.** Response curves of the main variables used to predict GIB potential distribution. The curves show how the logistic prediction changes with each environmental variable, keeping all other variables at their mean sample value. Red lines represent the average trend for the variable considered, while the blue shading represents the standard deviation from 1000 cross-validated replications.

**Cluster 1: Near Murtizapur village of Akola District of Maharashtra**

This cluster is represented by one 12 x 12 km grid covering an area of 144 km^2^ area. The identified priority conservation area falls near Murtizapur of Akola District. The overall land use is dominated by kharif crops followed by mixed crops. The areas under each land use and infrastructure feature is provided in the table, along with the corresponding map (Figure S2). GIB presence in this grid was confirmed to be within the last one month during the survey.

| Landuse Feature | Area % | Landuse Feature | Area % |
| --- | --- | --- | --- |
| Built-up | 1.51 | Plantation | 0.0 |
| Kharif Crop | 56.71 | Deciduous Forest | 1.31 |
| Rabi Crop | 2.31 | Degraded Forest | 0.08 |
| Zaid Crop | 0.0 | Wasteland | 1.83 |
| Double/Triple Crop | 16.70 | Water | 2.11 |
| Current Fallow | 17.44 | Mines | 0.0 |
| Infrastructure Feature | **Length (km)** | **Infrastructure Feature** | **Length (km)** |
| Railway track | 40.0 | Powerline | 242.1 |
| Roads | 73.8 | Canal | 0.0 |

**S2 Fig.** Priority conservation area identified during landscape survey in Akola District, Maharashtra, India

**Cluster 2: Near Arvi and Pimpalgaon of Wardha District of Maharashtra**

This cluster has one 12x12 km grid covering an area of 144 km^2^ area. This identified priority conservation area falls near Arvi and Pimpalgaon of Wardha District. The overall land use is dominated by kharif crops and mixed crops. The areas under each land use and infrastructure is provided in the table below, along with the corresponding map (Figure S3). GIB presence in this grid was confirmed to be within the last six months during the survey.

| Landuse Feature | Area % | Landuse Feature | Area % |
| --- | --- | --- | --- |
| Built-up | 0.5 | Plantation | 0.1 |
| Kharif Crop | 45.2 | Deciduous Forest | 11.2 |
| Rabi Crop | 1.4 | Degraded Forest | 1.4 |
| Zaid Crop | 0.0 | Wasteland | 11.1 |
| Double/Triple Crop | 12.8 | Waterbody | 2.4 |
| Current Fallow | 14.1 | Mines | 0.0 |
| Infrastructure Feature | **Length (km)** | **Infrastructure Feature** | **Length (km)** |
| Railway track | 0.0 | Powerline | 401.1 |
| Roads | 0.0 | Canal | 0.0 |

**S3 Fig.** Priority conservation area identified during landscape survey in Wardha District, Maharashtra, India

**Cluster 3: Near Warora and Wani villages of Chandrapur and Yawatmal District of Maharashtra**

This cluster has eleven 12x12 km grids covering an area of 1584 km^2^ area. This identified priority conservation area falls near Warora and Wani villages of Chandrapur and Yawatmal Districts. The overall land use is dominated by Kharif crops followed by mixed crops. The areas under each land use and infrastructure is provided in the table below, along with the corresponding map (Figure S4). GIB presence in this grid was confirmed to be within the last one month during the survey.

| Landuse Feature | Area % | Landuse Feature | Area % |
| --- | --- | --- | --- |
| Built-up | 2.5 | Plantation | 0.0 |
| Kharif Crop | 44.3 | Deciduous Forest | 6.7 |
| Rabi Crop | 0.9 | Degraded Forest | 3.1 |
| Zaid Crop | 0.0 | Wasteland | 8.9 |
| Double/Triple Crop | 18.8 | Waterbody | 2.2 |
| Current Fallow | 12.8 | Mines | 0.0 |
| Infrastructure Feature | **Length (km)** | **Infrastructure Feature** | **Length (km)** |
| Railway track | 52.8 | Powerline | 2349.1 |
| Roads | 330.1 | Canal | 89.7 |

**S4 Fig.** Priority conservation area identified during landscape survey in Chandrapur and Yawatmal, Maharashtra, India

**Cluster 4: Hingoli District**

This cluster has two 12x12 km grids covering an area of 288 km^2^ area. This identified priority conservation area falls near Hingoli of Hingoli District. The overall land use is dominated by Kharif crops followed by wastelands and Deciduous forests. The areas under each land use and infrastructure are provided in the table below along with the corresponding map (Figure S5). GIB presence in this grid was confirmed to be within the last one month during the survey.

| Landuse Feature | Area % | Landuse Feature | Area % |
| --- | --- | --- | --- |
| Built-up | 1.1 | Plantation | 0.3 |
| Kharif Crop | 30.1 | Deciduous Forest | 6.5 |
| Rabi Crop | 6.4 | Degraded Forest | 1.9 |
| Zaid Crop | 0.4 | Wasteland | 11.4 |
| Double/Triple Crop | 20.8 | Waterbody | 6.2 |
| Current Fallow | 14.9 | Mines | 0.0 |
| Infrastructure Feature | **Length (km)** | **Infrastructure Feature** | **Length (km)** |
| Railway track | 63.4 | Powerline | 86.6 |
| Roads | 0.0 | Canal | 13.0 |

**S5 Fig.** Priority conservation area identified during landscape survey in Hingoli District, Maharashtra, India

**Cluster 5: Near Loha village of Nanded District**

This cluster has one 12x12 km grid covering an area of 144 km^2^ area. This identified priority conservation area falls near Loha village of Nanded District. The overall land use is dominated by Kharif crops followed by wastelands and deciduous forests. The agriculture practice in the area is traditional. The areas under each land use and infrastructure are provided in the table below along with the corresponding map (Figure S6). GIB presence in this grid was confirmed to be within the last one month during the survey.

| Landuse Feature | Area % | Landuse Feature | Area % |
| --- | --- | --- | --- |
| Built-up | 2.1 | Plantation | 0.0 |
| Kharif Crop | 62.0 | Deciduous Forest | 0.0 |
| Rabi Crop | 0.0 | Degraded Forest | 0.0 |
| Zaid Crop | 0.0 | Wasteland | 3.3 |
| Double/Triple Crop | 30.4 | Waterbody | 1.6 |
| Current Fallow | 0.5 | Mines | 0.0 |
| Infrastructure Feature | **Length (km)** | **Infrastructure Feature** | **Length (km)** |
| Railway track | 0.0 | Powerline | 0.0 |
| Roads | 43.5 | Canal | 39.9 |

**S6 Fig.** Priority conservation area identified during landscape survey in Nanded Forest Division, Maharashtra, India

**Cluster 6: Ahmednagar District**

This cluster has six 12x12 km grids covering an area of 864 km^2^ area. This identified priority conservation area falls in the northeast direction of Ahmednagar city of Ahmednagar District. The overall land use is dominated by fallow lands followed by wasteland and major crops types are Kharif crops and Rabi crops. The areas under each land use and infrastructure is provided in the table below along with the corresponding map (Figure S7). GIB presence in this grid was confirmed to be within the last one month during the survey.

| Landuse Feature | Area % | Landuse Feature | Area % |
| --- | --- | --- | --- |
| Built-up | 2.2 | Plantation | 0.1 |
| Kharif Crop | 13.6 | Deciduous Forest | 2.8 |
| Rabi Crop | 5.0 | Degraded Forest | 3.8 |
| Zaid Crop | 0.0 | Wasteland | 17.0 |
| Double/Triple Crop | 3.7 | Waterbody | 1.1 |
| Current Fallow | 50.7 | Mines | 0.0 |
| Infrastructure Feature | **Length (km)** | **Infrastructure Feature** | **Length (km)** |
| Railway track | 242.0 | Powerline | 181.1 |
| Roads | 382.7 | Canal | 0.0 |

**S7 Fig.** Priority conservation area identified during landscape survey in Ahmadnagar District, Maharashtra, India

**Cluster 7: Near Patola and Borkhed of Beed District**

This cluster has one 12x12 km grid covering an area of 144 km^2^ area. This identified priority conservation area falls near Patola and Borkhed village of Beed District. The overall land use is dominated kharif crops followed by fallow lands. The agriculture practice in the area is traditional. The areas under each land use and infrastructure are provided in the table below, along with the corresponding map (Figure S8). GIB presence in this grid was confirmed to be within the last two months during the survey.

| Landuse Feature | Area % | Landuse Feature | Area % |
| --- | --- | --- | --- |
| Built-up | 0.6 | Plantation | 0.2 |
| Kharif Crop | 25.6 | Deciduous Forest | 1.8 |
| Rabi Crop | 2.5 | Degraded Forest | 6.1 |
| Zaid Crop | 0.0 | Wasteland | 26.0 |
| Double/Triple Crop | 5.3 | Waterbody | 1.7 |
| Current Fallow | 30.1 | Mines | 0.0 |
| Infrastructure Feature | **Length (km)** | **Infrastructure Feature** | **Length (km)** |
| Railway track | 0.0 | Powerline | 332.3 |
| Roads | 50.3 | Canal | 0.0 |

**S8 Fig.** Priority conservation area identified during landscape survey in Beed District, Maharashtra, India

**Cluster 8: Near Phaltan and Pimpari villages of Satara District**

This cluster has one 12x12 km grid covering an area of 144 km^2^ area. This identified priority conservation area falls near Phaltan and Pimpari villages of Satara District. The overall land use is dominated by fallow land followed by wasteland. The wasteland is without any powerline in the grid. The areas under each land use and infrastructure is provided in the table below along with the corresponding map (Figure S9). GIB presence in this grid was confirmed to be within the last one month during the survey.

| Landuse Feature | Area % | Landuse Feature | Area % |
| --- | --- | --- | --- |
| Built-up | 1.0 | Plantation | 0.0 |
| Kharif Crop | 3.7 | Deciduous Forest | 0.1 |
| Rabi Crop | 4.1 | Degraded Forest/ Forest | 1.4 |
| Zaid Crop | 0.0 | Wasteland | 34.1 |
| Double/Triple Crop | 1.2 | Waterbody | 1.5 |
| Current Fallow | 52.8 | Mines | 0.0 |
| Infrastructure Feature | **Length (km)** | **Infrastructure Feature** | **Length (km)** |
| Railway track | 0.0 | Powerline | 0.0 |
| Roads | 7.7 | Canal | 32.4 |

**S9 Fig.** Priority conservation area identified during landscape survey in Satara District, Maharashtra, India

**Cluster 9: Near Atpadi and Sangola villages of Sangli District**

This cluster has one 12x12 km grid covering area of 144 km^2^ area. This identified priority conservation area falls near Atpadi and Sangola of Sangli District. The overall land use is dominated by fallow land followed by kharif crops. The areas under each land use and infrastructure is provided in the table below, along with the corresponding map (Figure S10). GIB presence in this grid was confirmed to be within the last two months during the survey.

| Landuse Feature | Area % | Landuse Feature | Area % |
| --- | --- | --- | --- |
| Built-up | 1.2 | Plantation | 0.0 |
| Kharif Crop | 2.5 | Deciduous Forest | 0.0 |
| Rabi Crop | 8.4 | Degraded Forest | 0.0 |
| Zaid Crop | 0.1 | Wasteland | 13.1 |
| Double/Triple Crop | 1.6 | Waterbody | 2.7 |
| Current Fallow | 70.4 | Mines | 0.0 |
| Infrastructure Feature | **Length (km)** | **Infrastructure Feature** | **Length (km)** |
| Railway track | 0.0 | Powerline | 0.0 |
| Roads | 35.1 | Canal | 7.5 |

**S10 Fig.** Priority conservation area identified during landscape survey in Sangli District, Maharashtra, India

**Cluster 10: Near Mangalwedha and Nandeshwar of Solapur District**

This cluster has five 12x12 km grids covering an area of 720 km^2^ area. This identified priority conservation area falls near Mangalwedha and Nandeshwar in the southern part of Solapur District. The overall land use is dominated by fallow lands followed by Kharif crops and wastelands. The areas under each land use and infrastructure is provided in the table below along with the corresponding map (Figure S11). GIB presence in this grid was confirmed to be within the last six months during the survey.

| Landuse Feature | Area % | Landuse Feature | Area % |
| --- | --- | --- | --- |
| Built-up | 0.5 | Plantation | 0.1 |
| Kharif Crop | 4.9 | Deciduous Forest | 0.1 |
| Rabi Crop | 5.8 | Degraded Forest | 0.1 |
| Zaid Crop | 0.3 | Wasteland | 11.4 |
| Double/Triple Crop | 2.6 | Waterbody | 1.6 |
| Current Fallow | 72.6 | Mines | 0.0 |
| Infrastructure Feature | **Length (km)** | **Infrastructure Feature** | **Length (km)** |
| Railway track | 0.0 | Powerline | 219.7 |
| Roads | 367.6 | Canal | 236.3 |

**S11 Fig.** Priority conservation area identified during landscape survey in Solapur District, Maharashtra, India

**Cluster 11: Near Solapur, Tuljapur, Osmanabad, Latur and Ausa of Solapur, Osmanabad and Latur Districts**

This cluster has fifty-nine 12x12 km grid covering an area of 8496 km^2^ area and the largest area identified as priority conservation area. This identified priority conservation area covers the eastern part of the Solapur, the major part of Osmanabad District and western part of Latur District. The overall land use is dominated by Kharif crops followed by mixed crops. The areas under each land use and infrastructure are provided in the table below along with the corresponding map (Figure S12). GIB presence in this grid was confirmed to be within the last four days to 8 months during the survey.

| Landuse Feature | Area % | Landuse Feature | Area % |
| --- | --- | --- | --- |
| Built-up | 2.0 | Plantation | 0.2 |
| Kharif Crop | 20.3 | Deciduous Forest | 0.2 |
| Rabi Crop | 8.0 | Degraded Forest | 0.1 |
| Zaid Crop | 0.3 | Wasteland | 12.5 |
| Double/Triple Crop | 20.4 | Waterbody | 3.3 |
| Current Fallow | 32.6 | Mines | 0.0 |
| Infrastructure Feature | **Length (km)** | **Infrastructure Feature** | **Length (km)** |
| Railway track | 64.6 | Powerline | 1687.5 |
| Roads | 1944.1 | Canal | 647.7 |

**S12 Fig.** Priority conservation area identified during landscape survey in Solapur District, Maharashtra, India

**Cluster 12:** **Near Venkatapur of Adilabad District of Telangana**

This cluster has six 12x12 km grid covering an area of 864 km^2^ area. This identified priority conservation area falls near Venkatapur of Adilabad District. The overall land use is dominated by Kharif crops, deciduous forest and current fallow. The areas under each land use and infrastructure is provided in the table below, along with the corresponding map (Figure S13).

| Landuse Feature | Area % | Landuse Feature | Area % |
| --- | --- | --- | --- |
| Built-up | 2.2 | Plantation | 0.9 |
| Kharif Crop | 32.6 | Deciduous Forest | 20.9 |
| Rabi Crop | 0.4 | Degraded Forest | 4.0 |
| Zaid Crop | 0.0 | Wasteland | 8.6 |
| Double/Triple Crop | 4.6 | Waterbody | 5.7 |
| Current Fallow | 20.1 | Mines | 0.0 |
| Infrastructure Feature | **Length (km)** | **Infrastructure Feature** | **Length (km)** |
| Railway track | 0.0 | Powerline | 43.3 |
| Roads | 0.0 | Canal | 0.0 |

**S13 Fig.** Priority conservation area identified in Adilabad District, Telangana, India

**Cluster 13: Near Vikarabad of Rangareddi District of Telangana**

This cluster has nine 12x12 km grid covering an area of 1296 km^2^ area. This identified priority conservation area falls near Vikarabad village of Rangareddi District. The overall land use is dominated by mixed, Kharif crops and wastelands. The areas under each land use and infrastructure is provided in the table below, along with the corresponding map (Figure S14).

| Landuse Feature | Area % | Landuse Feature | Area % |
| --- | --- | --- | --- |
| Built-up | 3.3 | Plantation | 0.0 |
| Kharif Crop | 35.4 | Decidous Forest | 2.2 |
| Rabi Crop | 1.7 | Degraded Forest/ Forest | 0.9 |
| Zaid Crop | 0.0 | Wasteland | 8.5 |
| Double/Triple Crop | 37.7 | Waterbody | 2.5 |
| Current Fallow | 7.8 | Mines | 0.0 |
| Infrastructure Feature | Length (km) | Infrastructure Feature | Length (km) |
| Railway track in km | 95.0 | Powerline in km | 64.2 |
| Roads in km | 129.4 | Canal in km | 13.4 |

**S14 Fig.** Priority conservation area identified in Rangareddi District, Telangana, India

**Cluster 14: Near Thellarellapalli village of Mehbubnagar District of Telangana**

This cluster has nine 12x12 km grids covering an area of 1296 km^2^ area. This identified priority conservation area falls in Thellarellapalli area of Mehbubnagar District. The overall land use is dominated by Current fallow, Kharif crop, Rabi crops followed by wasteland. The areas under each land use and infrastructure are provided in the table below, along with the corresponding map (Figure S15).

| Landuse Feature | Area % | Landuse Feature | Area % |
| --- | --- | --- | --- |
| Built-up | 3.6 | Plantation | 1.2 |
| Kharif Crop | 26.0 | Decidous Forest | 2.4 |
| Rabi Crop | 11.2 | Degraded Forest/ Forest | 1.2 |
| Zaid Crop | 0.0 | Wasteland | 14.0 |
| Double/Triple Crop | 6.4 | Waterbody | 10.3 |
| Current Fallow | 23.9 | Mines | 0.0 |
| Infrastructure Feature | **Length (km)** | **Infrastructure Feature** | **Length (km)** |
| Railway track in km | 0.0 | Powerline in km | 159.4 |
| Roads in km | 119.0 | Canal in km | 188.4 |

**S15 Fig.** Priority conservation area identified in Mehbubnagar District, Telangana, India

**Cluster 15: Rollapadu Bird Sanctuary of Kurnool District of Andhra Pradesh**

This cluster has four 12x12 km grids covering an area of 576 km^2^ area. This identified priority conservation area falls in Rollapadu Bird Sanctuary, a protected area and an important GIB site of Kurnool District. The overall land use is dominated by Rabi crops, current fallow followed by wasteland. The areas under each land use and infrastructure is provided in the table below, along with the corresponding map (Figure S16).

| Landuse Feature | Area % | Landuse Feature | Area % |
| --- | --- | --- | --- |
| Built-up | 2.2 | Plantation | 0.0 |
| Kharif Crop | 12.1 | Decidous Forest | 0.0 |
| Rabi Crop | 30.2 | Degraded Forest/ Forest | 0.0 |
| Zaid Crop | 0.0 | Wasteland | 17.2 |
| Double/Triple Crop | 13.4 | Waterbody | 3.3 |
| Current Fallow | 21.5 | Mines | 0.0 |
| Infrastructure Feature | **Length (km)** | **Infrastructure Feature** | **Length (km)** |
| Railway track in km | 0.0 | Powerline in km | 104.7 |
| Roads in km | 16.7 | Canal in km | 55.8 |

**S16 Fig.** Priority conservation area identified in Rollapadu Bird Sanctuary in Kurnool District, Andhra Pradesh, India

**Cluster 16: Sirruguppa area of Bellary and Raichur Districts of Karnataka**

This cluster has eight 12x12 km grids covering an area of 1152 km^2^ area. This identified priority conservation area falls in Sirruguppa area of Bellary and Raichur District. The overall land use is dominated by Mixed, Kharif, Rabi crops, current fallow followed by wasteland. The areas under each land use and infrastructure is provided in the table below, along with the corresponding map (Figure S17).

| Landuse Feature | Area % | Landuse Feature | Area % |
| --- | --- | --- | --- |
| Built-up | 2.0 | Plantation | 0.3 |
| Kharif Crop | 24.6 | Decidous Forest | 0.2 |
| Rabi Crop | 10.1 | Degraded Forest/ Forest | 0.0 |
| Zaid Crop | 0.0 | Wasteland | 2.9 |
| Double/Triple Crop | 39.4 | Waterbody | 4.3 |
| Current Fallow | 16.2 | Mines | 0.0 |
| Infrastructure Feature | **Length (km)** | **Infrastructure Feature** | **Length (km)** |
| Railway track in km | 29.0 | Powerline in km | 132.0 |
| Roads in km | 204.6 | Canal in km | 52.3 |

**S17 Fig.** Priority conservation area identified in Sirruguppa area of Bellary and Raichur Districts of Karnataka
